# Numerosity Estimation in Visual Stimuli in the Absence of Luminance-Based Cues

**DOI:** 10.1371/journal.pone.0017378

**Published:** 2011-02-28

**Authors:** Peter Kramer, Maria Grazia Di Bono, Marco Zorzi

**Affiliations:** Dipartimento di Psicologia Generale, Università di Padova, Padova, Italy; University of Minnesota, United States of America

## Abstract

**Background:**

Numerosity estimation is a basic preverbal ability that humans share with many animal species and that is believed to be foundational of numeracy skills.

It is notoriously difficult, however, to establish whether numerosity estimation is based on numerosity itself, or on one or more non-numerical cues like—in visual stimuli—spatial extent and density. Frequently, different non-numerical cues are held constant on different trials. This strategy, however, still allows numerosity estimation to be based on a combination of non-numerical cues rather than on any particular one by itself.

**Methodology/Principal Findings:**

Here we introduce a novel method, based on second-order (contrast-based) visual motion, to create stimuli that exclude all first-order (luminance-based) cues to numerosity. We show that numerosities can be estimated almost as well in second-order motion as in first-order motion.

**Conclusions/Significance:**

The results show that numerosity estimation need not be based on first-order spatial filtering, first-order density perception, or any other processing of luminance-based cues to numerosity. Our method can be used as an effective tool to control non-numerical variables in studies of numerosity estimation.

## Introduction

It is widely believed that numeracy is founded upon a non-symbolic system of numerical representation (for reviews, see [1], [2]). At the heart of this system is the ability to perceive and discriminate numerosities (discrete quantities). This ability is predictive of math achievement [3]–[5], but children possess it before language acquisition [6], [7] and humans share it with various other species from monkeys [8]–[11] to fish [12].

The study of numerosity estimation is plagued by a fundamental problem: if we physically manipulate a collection of items to change its numerosity, then we not only change its numerosity, but inevitably also its physical dimensions. Take, for example, a collection of identical dots on a two-dimensional surface. If the numerosity of the collection is increased by adding another dot, then the collection must increase either in spatial extent (the area delineated by its outermost dots), or in density (interdot distance). It is unavoidable. A frequently recurring question in numerosity-estimation studies is therefore whether numerosity estimates are based on numerosity itself, or on one or more non-numerical cues like—in visual stimuli—spatial extent and density.

Although it is impossible to manipulate a numerosity and concurrently hold all non-numerical cues constant, many numerosity-estimation studies hold different non-numerical cues constant on different trials of the same experiment [8], [10], [13]. This way, it is hoped, subjects' numerosity estimates cannot be based on any of the non-numerical cues in particular, and will be based on numerosity itself. If, for example, only numerosity and spatial extent are manipulated on some of the trials, and only numerosity and density on the other trials, then neither spatial extent, nor density, can be used as a reliable cue to numerosity. Even if this procedure is followed, however, numerosity estimates need not rely on abstract numerosity. They could be based, concurrently, on more than one non-numerical cue, or—more parsimoniously—on a single combination of them [14], [15], [16], [17], [18]. These possibilities are much harder to control.

**Figure 1:**
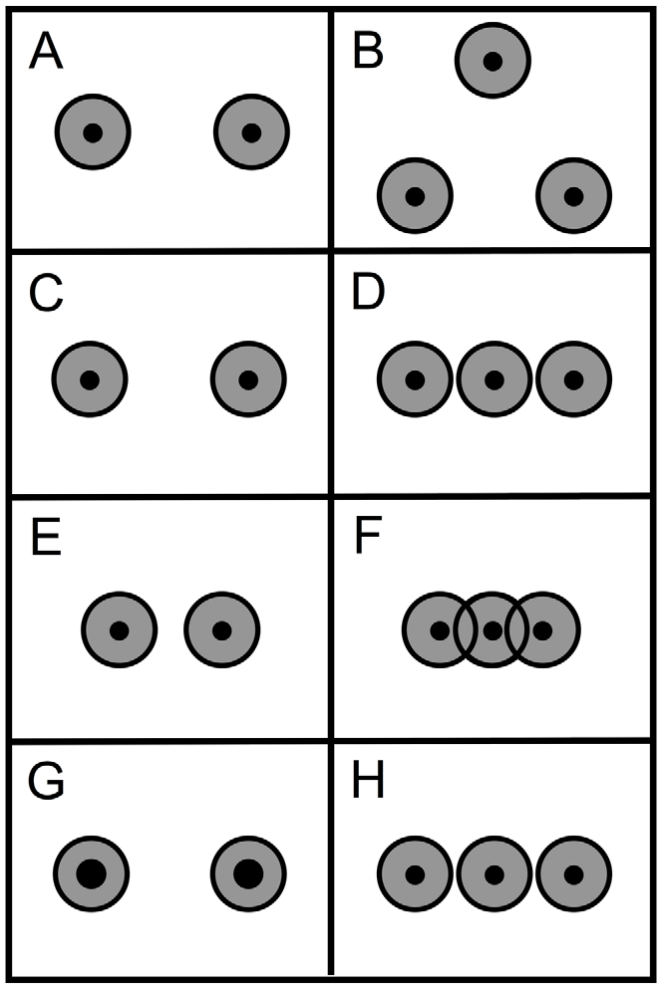
Influence spheres of numerosities containing either two or three items. The black dots have a numerosity of two in Panels A, C, E, and G and a numerosity of three in Panels B, D, F, and H. In Allik & Tuulmets's occupancy model [14], a numerosity estimate is given by the total area (*occupancy*) covered by the disk-shaped influence spheres (the set-theoretical union of the gray regions, including the black dots). From Panel A to Panel B, holding density constant, numerosity and occupancy are increased by increasing the collection's spatial extent. From Panel C to Panel D, holding spatial extent constant, numerosity and occupancy are increased by increasing the collection's density. From Panel E to Panel F, holding spatial extent constant, numerosity and occupancy are increased by increasing the collection's density, but due to the resulting overlap between the influence spheres, the occupancy is smaller in Panel F than in Panel D. From Panel G to Panel H, holding the combined surface area of the dots constant, numerosity and occupancy are increased by increasing the collections density. Thus, the model's numerosity estimate is the same in Panels A, C, E, and G and in Panels B, D and H. In Panels B, D, F, and H, it is larger than in Panels A, C, E, and G, but in Panel F it is smaller than in Panels B, D, and H. In Durgin's version of the model [17], [18], influence-sphere size decreases with dot density, and occupancy is normalized by dividing it by influence-sphere size.

Allik and Tuulmets, for example, presented a quantitative model of numerosity estimation of dot collections that is entirely based on a combined measure of the collections' spatial extent and density [14]. In this model, the authors assume that each dot is perceived with a surrounding disk-shaped influence sphere with a size that is the model's only free parameter (Figure 1). Human numerosity estimation is then predicted by the total area (*occupancy*) covered by the influence spheres. In the occupancy model, just as in humans, estimated numerosity follows a power function of actual numerosity [19]–[22], [ although cf. 23]. That is, ψ = c ϕ ^n^ , whereby ψ and ϕ represent psychological (estimated) numerosity and physical numerosity, and c and n represent constants (or alternatively: log(ψ) = n log( ϕ)+c). If numerosity is held constant, then in the occupancy model, just as in humans, estimated numerosity decreases with dot density [16], [20], [24]–[27] (compare Figures 1D and 1F), and is smaller for collections containing clusters of dots than for collections of evenly spaced ones [28]–[30].

It is important to note that occupancy is a combined measure of both spatial extent and density. If in an experiment only numerosity and spatial extent are manipulated on some of the trials (compare Figures 1A and 1B), and only numerosity and density on the other trials (compare Figures 1C and 1D, or Figures 1E and 1F, or Figures 1G and 1H), then numerosity and occupancy are manipulated on all trials. In principle, therefore, numerosity estimates in dot patterns could be based on occupancy, rather than on abstract numerosity, even if both spatial extent and density vary from trial to trial. Some studies control the combined surface area of the items [7], [13], [31] (as illustrated in Figures 1G and 1H). This surface area is sometimes called “occupied area” [13], but is unrelated to occupancy as defined in occupancy models.

Many studies investigate numerosity estimation in tasks in which one numerosity is compared to another. In some of these studies, the two numerosities are presented in different modalities (e.g., one in the visual, and one in the auditory, modality), or their items are presented simultaneously in one numerosity and sequentially in the other [32]–[35]. The occupancy model was not designed to handle such manipulations, and only applies to numerosities of simultaneously presented dots. The necessary extension of the model, however, is straightforward. When the dots are replaced by beeps, for example, and dispersed over time instead of space, then the influence spheres can be replaced by influence time-intervals. Occupancy is then the total time period covered by the influence intervals, and increases with the extent of this period and decreases with the rate of the beeps. There is indeed evidence that numerosity estimates decrease with rate in the temporal domain, just like they decrease with density in the spatial domain [36]. Crossmodal comparisons of numerosities can therefore, at least in principle, be based on crossmodal comparisons of occupancies rather than of abstract numerosities.

It has been argued that if abstract numerosities are represented in the brain, then numerosity comparisons across different modalities and modes of presentation should be as easy to perform as numerosity comparisons within the same modality and the same mode of presentation [32], [35]. If, instead, numerosities were represented as occupancies that are specific to modality and mode of presentation, then one would expect comparisons across these modalities and modes to be more difficult than comparisons within them. The data leave room for debate. Barth and colleagues [32], for example, found no effect of modality in their first experiment, and no effect of presentation mode in their second experiment, but did find a small effect of the combination of the two in their third experiment, and would have found it again in their fourth experiment if their test had been one-tailed instead of two-tailed (which would have been appropriate here, and while attempting to accept a null hypothesis, also more conservative). The authors report that, in a number of follow-up studies, the effects remained small, but that the comparisons across modalities and modes were nevertheless consistently more difficult than the comparisons within the same modality and mode. In macaque monkeys, rather than humans, Jordan and colleagues [35] did not find an effect of modality on accuracy, but did find that crossmodal numerosity comparisons were slower than intramodal ones. The results are thus rather mixed.

The most frequently used stimuli in numerosity-estimation studies do not involve different modalities or modes of presentation, but simultaneous presentations of collections of dots. For those stimuli, Allik and Tuulmets argue that occupancy can be computed on the basis of a simple spatial filtering of the stimulus [14]. Durgin rendered the occupancy model applicable to a wider range of numerosities by converting the model's constant influence-sphere size into one that decreases with density [17], [18]. He normalized the resulting occupancy by dividing it by influence-sphere size. Durgin too, though, argues that numerosity estimation is primarily a perceptual phenomenon. In his view, it is little more than a byproduct of density perception, which as its main purpose has the detection and recognition of textures and objects, and which—because the perceived density of a regular texture increases with distance—also plays a role in the perception of three-dimensional depth.

The numerosities, spatial filtering, and densities, considered by Allik and Tuulmets [14] and Durgin [17], [18], however, all involve luminance-defined items; the items are all either darker, or lighter, than their background. Although it is impossible to manipulate the numerosity of a physical collection of items and concurrently hold all its physical dimensions constant, it is possible to hold all its *luminance-defined* dimensions constant. In one of the stimuli that allow this to happen, the items move, but the motion is not *first-order* (luminance-based), but *second-order* (contrast-based [37]).

A popular method to obtain this second-order motion is to (1) create a region filled with black and white random dots (little squares), (2) consider an imaginary rectangular area within that region, (3) change all its black dots into white ones, and all its white dots into black ones, (4) laterally displace the imaginary area, and (5) repeat steps 3 and 4 several times. In a rapid presentation of the sequence, one sees the rectangular area in coherent second-order motion, even though the dots of which it consists do not show coherent first-order motion. Observers describe the moving rectangle as “transparent”, or “ghost-like”, whereas in any still image of the presentation it is undefined and undetectable. (For other kinds of second-order and higher-order motion, see [38]).

Here we introduce a new method based on second-order motion to create stimuli that exclude all first-order visual cues to numerosity. Our particular stimuli consist of gray and white dots, rather than black and white ones, and contain a variable number of rectangles that move back and forth in either first-order motion (in which case the rectangles are black, see Movie S1), or second-order motion (see Movie S2). The task is to estimate the numerosity of the rectangles. If the task can be performed in both motion conditions, then numerosity estimation cannot be based on first-order spatial filtering (as suggested by Allik & Tuulmets [14]), or first-order density perception (as suggested by Durgin [17], [18]), or on the perception of any other luminance-based cue to numerosity. If, instead, the task can only be performed in first-order motion, and not in second-order motion, then one would have to conclude that estimation of abstract numerosity does not exist. (In the current study, we only investigate the estimation of numerosities well beyond the so-called subitizing range of three or four items, but of course, our technique could be applied to very small numerosities too.).

## Results

### Means

**Figure 2:**
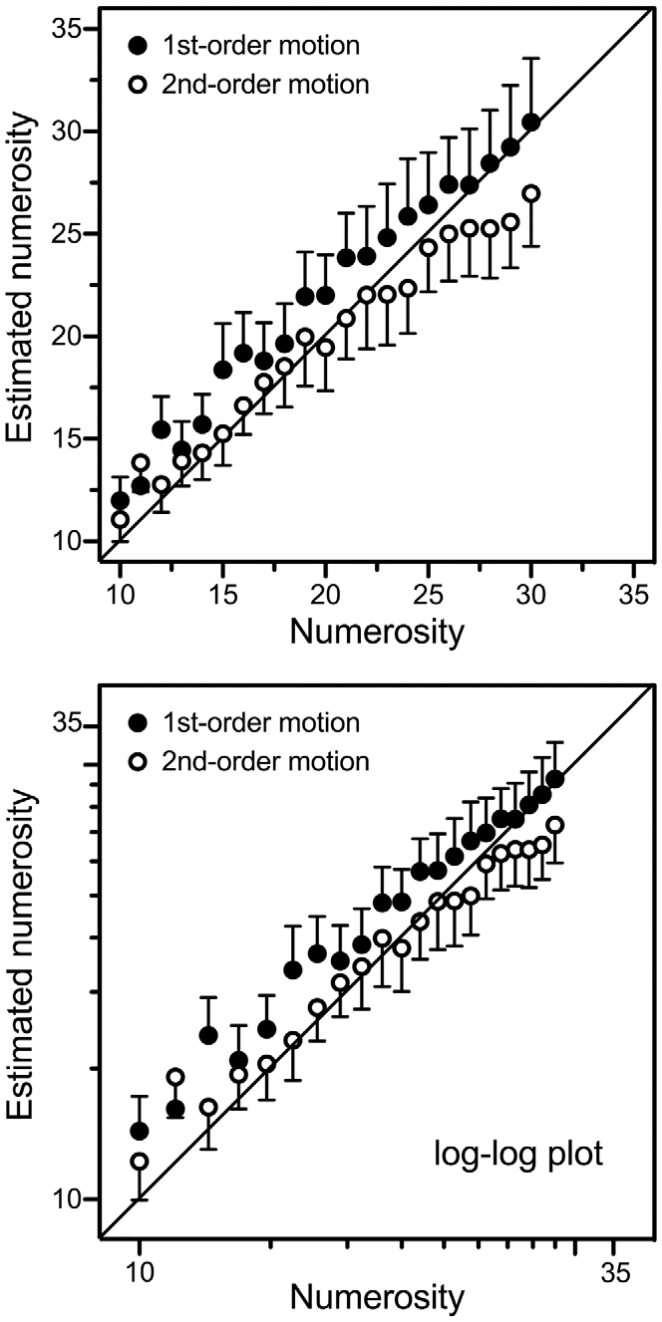
Numerosity estimation in first-order and second-order motion. A linear-linear plot (top panel), and a log-log plot (bottom panel), of estimated numerosity in first-order motion (filled symbols) and second-order motion (open symbols) as a function of actual numerosity (one subject was unable to do the task even in first-order motion and was excluded from the figures). Note that the error bars (representing one standard error of the mean) increase with numerosity in the linear-linear plot, but remain constant in the log-log plot. Note also, in the bottom panel, that the relationship between estimated and physical numerosity approximately follows the power law mentioned in the text: log(ψ) = n log( ϕ)+c.

One subject was neither able to perform the task in first-order motion, nor in second-order motion, and was excluded from the group analyses. For the first-order, and second-order, motion conditions (Figure 2, filled and open symbols, respectively), standard errors increased with estimated numerosity (respectively, adjusted R^2^ = .90 and adjusted R^2^ = .81, both *p*<.001). Instead, for both conditions, the coefficients of variation (i.e., standard deviation/mean) of the numerosity estimates were unrelated to the estimates themselves (for both conditions R^2^<.01, both with coefficient means of .33). These results suggest that the standard errors increased in direct proportion to the numerosity estimates (*scalar variability*), which indicates that subjects were indeed estimating rather than counting [39], [40]. We confirmed that this was indeed the case, with two separate regression analyses for the first- and second-order-motion conditions, on the logarithmically transformed data. As required [13], [39], with numerosity estimation as the independent and standard error as the dependent variable, the regression slopes for the two conditions were both close to one (r = .96 and r = .93).

A repeated-measures ANOVA on the logarithmically transformed data revealed a main effect of motion order (*F*(1,10) = 41.43, *p*<.001, η_p_^2^ = .81); numerosity estimates were higher in first-order, than in second-order, motion (Figure 2, bottom panel). There was also a main effect of numerosity (*F*(20,200) = 63.76, *p*<.001, η_p_^2^ = .86); as numerosity increased so did the estimates. In addition, there was an interaction between motion order and numerosity (*F*(20,200) = 1.92, *p* = .01, η_p_^2^ = .16); the difference in numerosity estimation between the first- and second-order motion conditions was slightly larger at the high, than at the low, numerosities (Figure 2). Results changed little when the subject who was unable to perform the task even in first-order motion was included in the analysis.

Figure 2 suggests that, in first-order motion, all numerosities were overestimated with an amount that decreased with numerosity. Figure 2 also suggests that, in second-order motion, small numerosities were overestimated, and large ones underestimated. For no particular numerosity, though, did any over- or underestimation reach significance.

### Individual data

Averaging across subjects may potentially obscure important individual differences, and hence, we also examined the individual data. Given that item size has sometimes been found to affect numerosity estimation [16], [26], [41], we also investigated the potential effects of the differences in average bar height between stimuli with the same numerosity (bar width did not vary). Bars that differ in height should also be expected to activate motion-sensitive receptive fields of different sizes, and for this reason too, the potential effect of bar height needs to be investigated.

For each subject, we performed one multiple regression for the first-order-motion data (Table 1) and one for the second-order-motion data (Table 2) with numerosity and bar height as the independent variables and estimated numerosity as the dependent variable. As can be seen in Tables 1 and 2, the two multiple correlations were both significant for eleven of the twelve subjects. For one subject, neither multiple correlation was significant. These results overwhelmingly confirm that numerosity estimation is possible, not just in first-order, but also in second-order, motion.

**Table 1:** Multiple regression results per subject: First-order-motion condition.

Correlations		Standardized coefficients					
Adj. R^2^	*p*	log(num.)	*t*(125)	*P*	log(height)	t(125)	*p*
.76	<.01	0.96	3.14	<.01	0.09	−0.30	.77
.56	<.01	0.81	2.01	.05	−0.05	−0.14	.89
.49	<.01	0.94	2.12	.04	−0.23	−0.53	.60
.34	<.01	0.70	1.36	.18	−0.10	−0.19	.85
.77	<.01	0.57	1.95	.05	0.31	1.07	.29
.40	<.01	1.44	2.64	<.01	−0.81	−1.49	.14
.62	<.01	1.13	2.93	<.01	−0.34	−0.89	.38
.77	<.01	0.39	1.27	.21	0.49	1.58	.12
.32	<.01	0.48	0.97	.33	0.10	0.19	.85
.56	<.01	0.53	1.31	.19	0.23	0.56	.58
.76	<.01	0.74	2.28	.03	0.14	0.43	.67
.02	1.12	0.85	1.29	.20	−0.70	−1.06	.29

*Note.* Adj. R^2^ = Adjusted R^2^, log(num.) = log(estimated numerosity), t(125) = t-test with degrees of freedom, log(height) = log(bar height).

**Table 2:** Multiple regression results per subject: Second-order-motion condition.

Correlations		Standardized coefficients					
Adj. R^2^	*p*	log(num.)	*t*(125)	*P*	log(height)	t(125)	*p*
.73	<.01	1.21	3.57	<.01	−0.36	−1.06	.29
.55	<.01	0.13	0.29	.77	0.62	1.43	.16
.46	<.01	0.37	0.73	.46	0.32	0.64	.52
.39	<.01	1.79	3.70	<.01	−1.20	−2.47	.02
.72	<.01	0.72	2.22	.03	0.13	0.39	.69
.73	<.01	0.94	2.81	<.01	−l.09	−0.26	.79
.62	<.01	0.93	2.40	.02	−0.14	−0.36	.72
.81	<.01	0.82	3.00	<.01	0.08	0.29	.77
.34	<.01	0.92	1.62	.11	−0.33	−0.59	.56
.61	<.01	1.60	3.94	<.01	−0.82	−2.03	.04
.77	<.01	1.11	3.88	<.01	−0.23	−0.81	.42
−.001	.39	0.35	0.48	.63	−0.23	−0.32	.75

*Note.* Adj. R^2^ = Adjusted R^2^, log(num.) = log(estimated numerosity), t(125) = t-test with degrees of freedom, log(height) = log(bar height).

As can be seen in Tables 1 and 2, for none of the subjects did bar height have a significant positive coefficient in the regression equation, and for two of them it had a significant negative coefficient in the second-order motion condition. Thus, bar height was not a good predictor of numerosity estimation, in neither first-order, nor second-order, motion. In contrast, in both conditions, numerosity had a significant positive coefficient in the regression equation for seven of the eleven subjects that were able to perform the task in the first-order-motion condition, and for eight of them in the second-order-motion condition. For none of the subjects did numerosity have a negative coefficient. When bar height was removed from the regression equation, the simple correlation between numerosity and estimated numerosity in second-order motion was significant for all the eleven subjects that were able to do the task in first-order motion, with correlations ranging from r = .58 to r = .88 (all *p*<.001).

For each of the eleven subjects mentioned above, we calculated the average across the six estimates of each numerosity in both first-order, and second-order, motion and calculated the correlation between the two motion conditions for each subject separately. We found correlations ranging from r = .76 to r = .97 (all *p*<.001), indicating that estimates in the two motion-order conditions were quite similar.

## Discussion

We introduced a new method that, for the first time, using second-order motion, *concurrently* eliminated all luminance-based cues to numerosity in visual stimuli. We found that numerosity estimation in second-order motion is not only possible, but also only slightly different from that in first-order motion. Our results show that numerosity estimation need neither be based on occupancy (as it has been defined thus far), nor be affected by luminance-based cues to numerosity. They also show that numerosity estimation need neither rely on first-order spatial filtering (as suggested by Allik & Tuulmets [14]), nor on first-order density perception (as suggested by Durgin [17], [18]).

The small difference in performance that was observed between the two motion-order conditions may be due to the fact that the visual system is less sensitive to second-order, than to first-order, motion [42]. Our centrally presented items were well above perceptual threshold, but because sensitivity decreases with eccentricity, some peripheral items might not have been, and the probability of that should have been largest for the items defined in second-order motion. Because, in absolute numbers, there were more peripheral items in our large numerosities than in our small ones, the visual system's inferior sensitivity to second-order motion might have caused the interaction that we observed between motion order and numerosity. The size of the effect is small, though, and still allows the conclusion that numerosity estimation is not only possible in second-order motion, but also quite similar to that in first-order motion.

As is, the occupancy models of Allik and Tuulmets [14] and Durgin [17], [18] cannot explain the data we obtained in our second-order-motion condition, and neither can other models based on first-order cues [15], [16]. Let us examine in detail, though, the most recent of this class of models—the occupancy model by Durgin—and consider the possibility that it could be revised to explain our data. In the original occupancy model of Allik and Tuulmets influence spheres are defined around dots and have a fixed size. Durgin showed that although this model predicts numerosity estimation of dot collections very well at low densities, it must necessarily fail at either high, or low, densities. In Durgin's revision, the size of the influence spheres decreases with density, and occupancy is normalized by dividing it by influence-sphere size. According to Durgin, the perceptual system avoids an information overload by processing many items such as dots not as distinct entities, but as integral parts of textures (for related ideas, see also [43]). The perceptual system would create the textures by computing summary statistics, such as the mean and variance of item density. Subsequently, numerosity estimates would be based on these statistics rather than on individual items (see also [44], [45]).

That summary statistics are indeed computed is suggested by the fact that we see collections of items extrapolated (*filled-in*) into areas that do not receive any input (such as the retinal blindspot and scotomas), or into areas that are weakened by adaptation [46], [47]. In addition, it has been shown that, at least under some conditions, subjects can determine the average size of items better than their individual sizes [48], [49] and the average location of items better than their individual locations [50]. Durgin himself considers a large collection of dots and a copy of it from which, at random, many dots are removed. He demonstrates that it is easy to see that the two collections differ in density (as density information is retained), but difficult to see that the latter is a subcollection of the former (as, after the computation of the summary statistics, the information about the positions of the individual items would be lost).

Franconeri and colleagues [41] and He and colleagues [51] challenged whether numerosity estimation would be entirely based on the summary statistics of an unsegmented scene. Independently, both studies found that numerosity estimates are lower when items are connected by thin lines into larger perceptual objects than when these connections are severed by small gaps. The authors argue that the small gaps should not have affected the items' summary statistics much, and hence, that numerosity estimation can be affected by the segmentation of items into larger objects. In the connected condition, as a result of Stroop-like interference [16], [52], [53], the small number of task-irrelevant objects could have decreased the numerosity estimates of the task-relevant items. Both Franconeri and colleagues and He and colleagues conclude that numerosity estimation need not be based on summary statistics computed over unsegmented scenes.

In the studies of both Franconeri and colleagues [41] and He and colleagues [51], however, there are more line terminations in the unconnected, than in the connected, condition. Line terminations are texture elements (for a review, see [54]). It is possible that the perceptual system computes summary statistics across both the task-relevant items and the task-irrelevant line terminations. Numerosity estimates might then be biased upwards in the unconnected condition, as a result of Stroop-like interference between line terminations and items, rather than downwards in the connected condition, as a result of Stroop-like interference between perceptual objects and items. The two studies, thus, do not exclude the possibility that numerosity estimation could be entirely based on summary statistics.

The question, though, is whether a revised occupancy model could explain our present data. We think that it is possible in principle, but that a revision would face three problems. First, if the new model, like Durgin's, is to adjust influence-sphere size on the basis of summary statistics, then it needs to be established whether the perceptual system computes these summary statistics across items defined in second-order motion, and whether it uses these statistics to adjust influence spheres. The computation and adjustment might be challenging with stimuli like ours in which items are presented for only 133 ms, afterwards immediately masked by the random-dot pattern of the background, and invisible in any still image of the motion.

Second, although the density of our items was the same across our motion-order conditions, the numerosity estimates were not. The difference between the estimates was small, but because it was significant, the model would have to take it into account. That is, the model would have to be redefined in such a way that its influence spheres would not only depend on density, but also on motion order (or item type).

Third, as their numerosity increases, visually presented items are forced either further into the periphery, or closer together. Under both these conditions, even if the items remain visible, their discriminability diminishes (*crowding* [55]). If the diminished discriminability were to affect numerosity estimation, then one would expect large numerosities to be underestimated and the underestimation to increase with numerosity. Indeed, many studies have found both these effects (e.g., [13], [14]). A kind of discriminability is modeled, in the occupancy models, by the size of the influence spheres: the larger the influence spheres of two items, the less they count as two, and the more they count as just one. The discriminability of items, however, is a complex matter (for a special issue on just crowding, for example, see the Journal of Vision, 7(2)). It remains to be seen whether a revised occupancy model could adequately account for the distinguishability of items in general rather than only of luminance-defined dots. More philosophically, one may wonder whether the distinguishability of items has anything to do with numerosity estimation, or whether it may only concern its prerequisites [16].

A density low enough to ensure that all items can be distinguished from each other appears to be a prerequisite for optimal numerosity estimation. It thus makes sense that density affects numerosity estimation, even if numerosity estimation is not based on density perception. In simultaneously presented collections, numerosity estimates increase with the frame size of the display [56], and decrease with item size [57], [58], [59], [cf. 60] (which might increase perceived display size [41]). These effects do not concern prerequisites to numerosity estimation. Franconeri et al., however, argue that the non-numerical quantities might be processed separately from the numerical ones and create Stroop-like interference only at a late response selection stage [41]. Indeed, this possibility suggests that interactions between numerical and non-numerical factors by themselves cannot be taken as evidence that the former must be based on the latter.

In conclusion, we have shown that numerosities can be estimated almost as well in second-order motion as in first-order motion, despite the *concurrent* exclusion of all first-order cues to numerosity. The result shows that numerosity estimation need neither be based on first-order spatial filtering, nor on first-order density perception. No perceptual model of numerosity estimation can account for our data, and any revisions face three major challenges. The best way forward may be to separate the issue of item-discriminability from that of numerosity estimation itself. In any event, our method of creating stimuli that exclude all first-order visual cues to numerosity can be used as an effective tool to control non-numerical variables in studies of numerosity estimation.

## Materials and Methods

### Ethics Statement

The data were analyzed anonymously. Subjects verbally provided their informed consent and confirmed it in writing by signing a form to receive compensation for participating. The current study has been approved by the ethics committee of the Department of General Psychology of the University of Padova.

### Subjects

Twelve naïve undergraduates (aged 22–27, 4 women) of the Università di Padova participated for a small monetary reward.

### Apparatus

An IBM-compatible computer with a 17″ flat-screen monitor (75 Hz refresh rate; 1024×768 resolution), and a custom E-Prime program (Psychology Software Tools, Inc.), were used for the millisecond-precise stimulus presentation. Viewing distance was 58 cm, controlled with a chin-and-head rest.

### Stimuli

The stimuli contained a number of bars (vertically oriented rectangles) that ranged from 10 through 30. The background, visible throughout the experiment, consisted of random gray and white little squares (4×4 pixels) that filled the screen. The bars were each equally likely to be either 0.13°×2.08° or 0.13°×3.12° large, and in half the trials they were black (first-order-motion condition), and in half the trials they consisted of little squares identical to those in the background (second-order-motion condition). At the start of each trial, the bars all moved 1.84° to the right, then 1.84° back to the left, and then disappeared. They were visible for 133 ms, short enough to prevent subjects from counting them, or inspecting them with multiple saccades (note that information integration across saccades is imperfect [61]). Each rectangle appeared at least 1.84° away from any other rectangle to the left or right of it, and at least 0.98° away from any rectangle above or below it. Within these restrictions, the rectangles appeared randomly in one of the cells of a 17×12 imaginary grid.

### Design and procedure

Both in the first-order-motion, and second-order-motion, condition subjects were provided with three “calibration” trials in which the numerosity of twenty was shown, along with a red numeral 20 in the lower-left corner of the screen. Such “calibration” trials can improve subsequent estimates of even those numerosities for which feedback is never provided, and also serve to reduce inter-subject variability [13]. After the calibration trials, forty practice trials were presented, without feedback, containing numerosities randomly chosen from the range 10 through 30. Next, the experiment proper started, which did not provide feedback either. It had a completely randomized within-subjects design. After each trial, following Izard and Dehaene [13], subjects responded by typing a number on the keyboard corresponding to their numerosity estimate. In the experiment proper, each numerosity was presented six times, and after each trial subjects advanced to the next by pressing the space bar.

## Supporting Information

Movie S1 **Numerosity defined in first-order motion.**
(AVI)

Movie S2 **Numerosity defined in second-order motion.**
(AVI)
